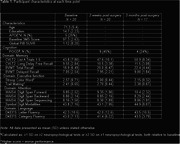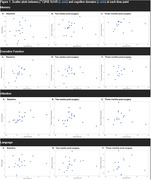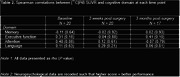# The Association between Brain Amyloid‐Beta Deposition and Postoperative Cognitive Dysfunction After Pelvic Organ Prolapse Surgery: A Pilot Study

**DOI:** 10.1002/alz.095383

**Published:** 2025-01-09

**Authors:** Mary F Ackenbom, Sarah K. Royse, Esa M. Davis, Steven R. Orris, Laura G. Vargas, Meryl A Butters

**Affiliations:** ^1^ Magee‐Womens Research Institute, Pittsburgh, PA USA; ^2^ University of Pittsburgh, Pittsburgh, PA USA; ^3^ University of Maryland, Baltimore, MD USA; ^4^ University of Pittsburgh Medical Center, Pittsburgh, PA USA; ^5^ University of Pittsburgh School of Medicine, Pittsburgh, PA USA

## Abstract

**Background:**

Research on the role of preclinical AD pathology (brain Aβ deposition) and postoperative cognitive dysfunction is limited. We aimed to explore the association between brain Aβ deposition measured via Pittsburgh Compound B (PiB) PET imaging and POCD. We hypothesized that brain Aβ deposition would be associated with POCD in older women at 2 weeks and 3 months after surgery.

**Method:**

This is a prospective cohort study of women aged ≥60 years who underwent urogynecologic surgery. Exclusion criteria included cognitive impairment diagnosis, abnormal cognition screen (Modified Mini‐mental State Exam (3MS) score < 84), and history of stroke. Baseline clinical and sociodemographic data were ascertained by interview and chart review. Brain Aβ deposition (global PiB standardized uptake value ratio (SUVR)) was measured through PiB‐PET using a combined MR/PET scanner. Eight neuropsychological tests, assessing memory, executive function, attention, and language were administered preoperatively, 2 weeks post‐surgery and 3 months post‐surgery. POCD was defined as ≥1 standard deviation (SD) decline on ≥2 neuropsychological tests or ≥ 2 SD decline on ≥1 tests. The association between Aβ PiB SUVR and cognitive performance was tested with Spearman correlation coefficient. Linear regression models, adjusted for clinically relevant variables, were generated.

**Result:**

There were 20 patients enrolled with mean±SD age of 71.3±5.4 years, 14.7±2.23 years of education, baseline 3MS score of 97.3±2.63, and 1.12±0.20 global PiB SUVR. There was 45% (n = 9) POCD incidence at 2 weeks postoperatively and at 24% (n = 4/17) POCD prevalence at 3 months postoperatively. Table 1 summarizes demographics and cognitive performance at each time point. There were no significant correlations between PiB and cognitive domain at each time point although there appears to be a trend towards association of Aβ deposition on Spearman correlations (Table 2; Figure 1). On exploratory linear mixed models, there were no significant associations between Aβ deposition and cognitive performance by domain, nor with POCD.

**Conclusion:**

In this cohort of older women, 45% had POCD 2 weeks post‐surgery and 24% had POCD 3 months post‐surgery. Brain Aβ deposition was not associated with cognitive performance nor POCD. Studies with larger cohorts are needed to fully investigate this potential predisposing risk factor.